# Elevated Blood Urea Nitrogen is Associated With Critical Limb Ischemia in Peripheral Arterial Disease Patients

**DOI:** 10.1097/MD.0000000000000948

**Published:** 2015-06-19

**Authors:** Thomas Gary, Martin Pichler, Gernot Schilcher, Franz Hafner, Gerald Hackl, Peter Rief, Philipp Eller, Marianne Brodmann

**Affiliations:** From the Division of Angiology (TG, FH, GH, PR, PE, MB); Division of Oncology (MP); and Division of Nephrology (GS), Department of Internal Medicine, Medical University Graz, Graz, Austria.

## Abstract

As renal function is often impaired in atherosclerosis patients, accelerating atherosclerosis per se and creating a vicious cycle, we investigated the association of blood urea nitrogen (BUN) and critical limb ischemia (CLI) in peripheral arterial occlusive disease (PAOD) patients.

Our cross-sectional study included 1521 PAOD patients, with normal and impaired renal function treated at our institution from 2005 to 2010. Patients on renal replacement therapy were excluded. The cohort was divided into tertiles according to the serum BUN levels. An optimal cutoff value for the continuous BUN was calculated by applying a receiver-operating curve analysis to discriminate between CLI and non-CLI.

In our cohort, CLI increased significantly with an increase in BUN (13.1% in the first tertile, 18.7% in the second tertile, 29.0% in the third tertile, *P* for trend <0.001). A BUN of 17.7 mg/dL was identified as an optimal cutoff. Accordingly, there were 2 groups of patients: 636 patients with BUN ≤ 17.7 and 885 patients with BUN > 17.7. CLI was more frequent in BUN > 17.7 patients (342 [38.6%]) than in BUN ≤ 17.7 patients (134 [21.1%]) (*P* < 0.001); the same applied to prior myocardial infarction (45 [5.1%] vs 15 [2.4%], *P* = 0.007) and congestive heart failure (86 [9.7%] vs 31 [4.9%], *P* < 0.001). A BUN > 17.7 was associated with an odds ratio of 1.6 (95% confidence interval: 1.3–1.9, *P* < 0.001) for CLI even after the adjustment for other established vascular risk factors such as age ≥75 and type 2 diabetes.

An increased BUN is significantly associated with a high risk for CLI and other vascular endpoints. The BUN is an easily determinable, broadly available, and inexpensive marker that could be used to identify patients at high risk for vascular endpoints.

## INTRODUCTION

Renal impairment is associated with vascular endpoints in atherosclerosis patients, and recent publications have shown an association of uric acid levels with adverse cardiovascular disease (CVD) outcomes in patients with stable coronary heart disease (CHD).^1^ One report showed that uric acid levels may predict future cardiovascular endpoints in patients with stable CVD, and that risk is increased even in patients with uric acid levels currently considered normal.^1^

Serum blood urea nitrogen (BUN) is a further parameter mainly influenced by renal function. Studies of BUN in CHD patients have revealed a close association between long-term mortality and acute ST-elevation myocardial infarction (STEMI).^2^ The authors argue that BUN could be a useful parameter to reflect hemodynamic alterations and neurohumoral activation in these patients.

Peripheral arterial occlusive disease (PAOD) is a highly prevalent but underdiagnosed manifestation of atherosclerosis.^3^ If PAOD is not diagnosed timely and treatment initiated, disease progression with the development of critical limb ischemia (CLI) is highly probable.^4^ CLI is an entity with high mortality and high risk of limb amputation. Although treatment options, including endovascular procedures, have improved in recent years, mortality and amputation rates remain high.^5,6^

We investigated the association of BUN with CLI in PAOD patients. As BUN seems to reflect hemodynamic changes in atherosclerosis patients, it might be a suitable parameter to identify PAOD patients whose low perfusion suggests high risk for CLI. Patients on renal replacement therapy were excluded from our analysis as serum BUN levels do not reflect their degree of renal impairment.

## METHODS

Our cross-sectional study included 1521 successive PAOD patients, with normal and impaired renal function treated at our institution from 2005 to 2010. The only exclusion criterion was current renal replacement therapy. The study was approved by the International Review Board of the Medical University of Graz, Austria. As this was a retrospective data analysis of blinded data, the ethics committee waived written consent.

According to the TASC II criteria, the diagnosis of PAOD was based on clinical evaluation, ankle brachial index (ABI), and duplex scan. Severity of disease was assessed with the Fontaine classification. According to current guidelines and corresponding to Fontaine classes 3 and 4, PAOD was defined in CLI patients when they presented with ischemic rest pain and/or skin ulceration/gangrene.^7^ Upon admission, patients responded to a standardized questionnaire with attention to cardiovascular risk factors and comorbidities. Clinical symptoms were evaluated in the course of the physical examination. Blood for laboratory work was drawn from fasting patients.

### Statistical Analyses

Clinical characteristics were analyzed using descriptive statistics. For comparison of groups, the χ^2^ test for categorical values, *t* test for normally distributed continuous variables, and Mann–Whitney *U* test for nonnormally distributed continuous variables were used.

The study population was divided into tertiles according to their continuous BUN starting with the lowest BUN. Occurrence of CLI in BUN tertiles was evaluated. A Jonckheere–Terpstra test was performed to reveal any statistical trend for BUN and CLI. The optimal cutoff value for the continuous BUN was calculated by applying a receiver-operating curve analysis to test all possible cutoffs that would discriminate between CLI and non-CLI.

We further calculated odds ratios (ORs) with 95% confidence intervals (CIs) for different CLI risk factors, including BUN with a binary logistic regression model. All tests used a *P* value of 0.05 as threshold for significance. SPSS 17.0 was used for all statistical analyses.

## RESULTS

**FIGURE 1:**
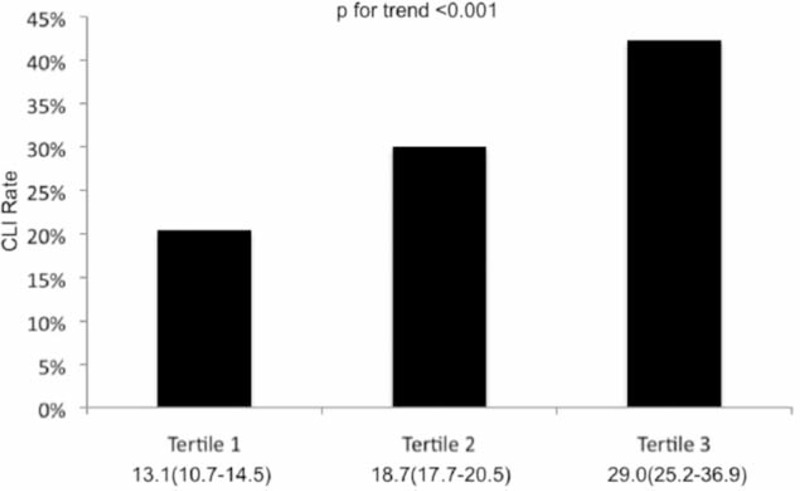
Percentage of patients with critical limb ischemia stratified by tertiles of blood urea nitrogen. Numbers below the figure are median blood urea nitrogen and the 25th and 75th percentile.

The characteristics of the 1521 PAOD patients are shown in Table 1. In a first step, on the basis of their BUNs, the study population was divided into tertiles of 507 patients each. CLI increased significantly with an increase in BUN (13.1% in the first tertile, 18.7% in the second tertile, 29.0% in the third tertile; Figure 1). A Jonckheere–Terpstra test was performed to evaluate the trend for increase in CLI rate with increasing BUN showed statistical significance (*P* < 0.001).

**TABLE 1:**
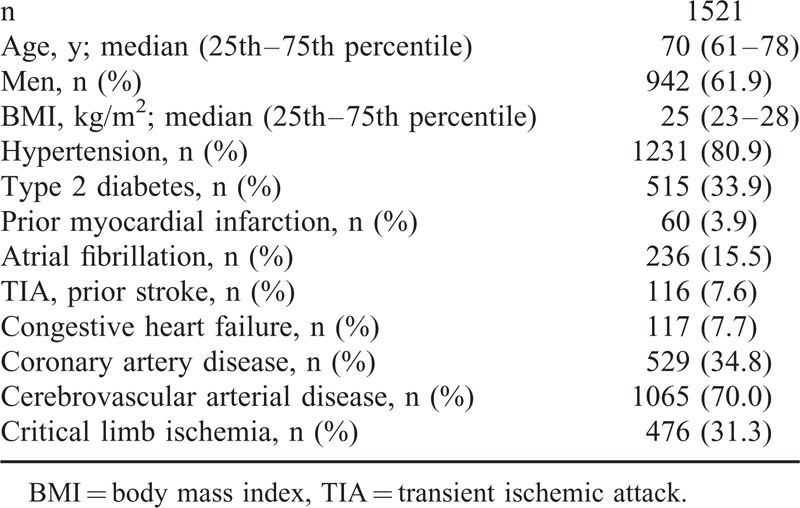
Patients’ Characteristics of All Peripheral Arterial Occlusive Disease Patients Included in the Study

In a second step, a BUN of 17.7 mg/dL was identified through a receiver-operating curve analysis as an optimal cutoff value to discriminate between CLI and non-CLI. Two groups were formed accordingly: one with 636 patients (BUN ≤ 17.7) and the other with 885 patients (BUN > 17.7) (see Table 2). Both groups also differed significantly in terms of other parameters of renal function, like creatinine and glomerular filtration rate (GFR), calculated with the Chronic Kidney Disease Epidemiology Collaboration (CKD-EPI) GFR Estimation.^8^ Concerning vascular endpoints CLI was more frequent in BUN > 17.7 patients (342 [38.6%]) than in BUN ≤ 17.7 patients (134 [21.1%]) (*P* < 0.001), as was also true for prior myocardial infarction (45 [5.1%] vs 15 [2.4%] *P* = 0.007) and congestive heart failure (86 [9.7%] vs 31 [4.9%], *P* < 0.001).

**TABLE 2:**
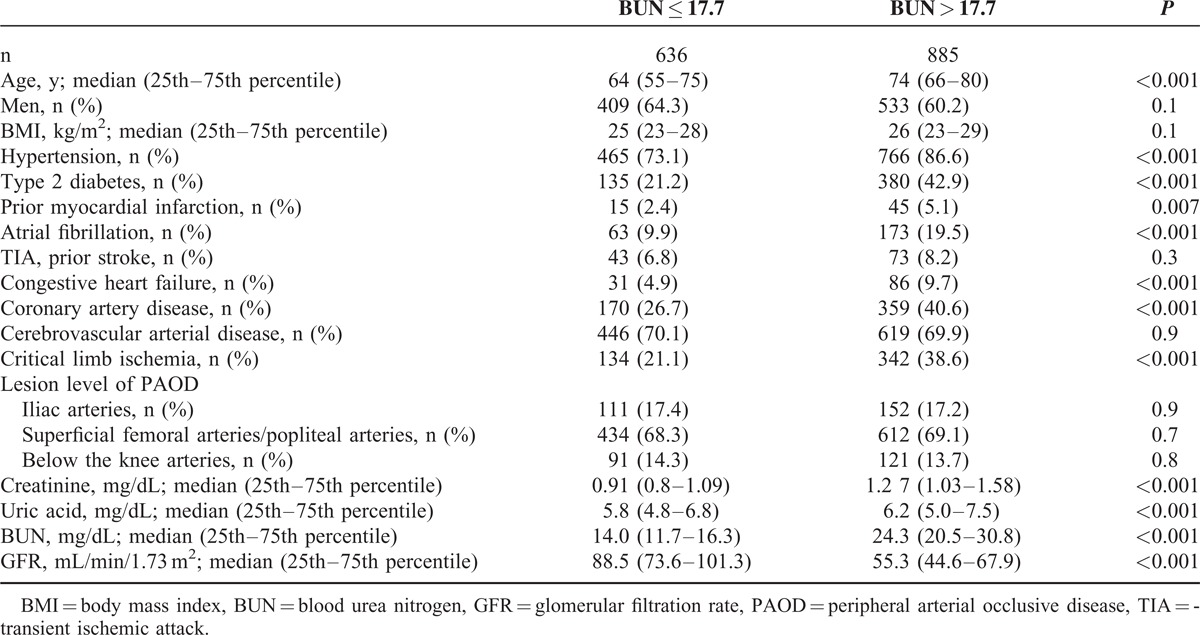
Clinical and Hematological Characteristics of Population With BUN ≤17.7 and >17.7

In a third step, BUN > 17.7 was used as a variable in a binary logistic regression model to evaluate it as an independent risk factor for CLI. We further included sex, type 2 diabetes, age >75, coexistence of congestive heart failure, and history of stroke/transient ischemic attack. A BUN > 17.7 was associated with an OR of 1.6 (95% CI: 1.3–1.9, *P* < 0.001) for CLI even after the adjustment for other established vascular risk factors (see Table 3).

**TABLE 3:**
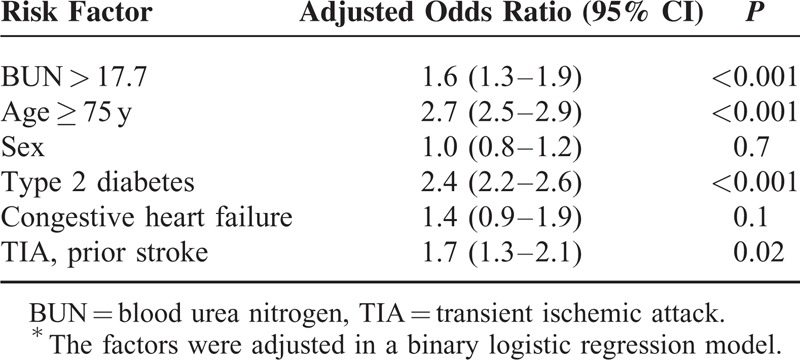
Adjusted Risk Factors for Critical Limb Ischemia in Peripheral Arterial Occlusive Disease Patients^∗^

## DISCUSSION

We were able to demonstrate that BUN > 17.7 is associated with a high risk for CLI in PAOD patients. Even after the adjustment for other main CLI risk factors such as diabetes and advanced age (>75), BUN > 17.7 was associated with a 1.6-fold increase in CLI risk. Not only CLI was found more frequently in the high BUN group, but also endpoints because of atherosclerotic lesions in other vascular beds, like myocardial infarction, were more frequent. Even cardiac entities associated with coronary artery disease, like congestive heart failure and atrial fibrillation,^9^ were significantly more prevalent. Congestive heart failure might also worsen CLI symptoms because of low cardiac output. However, even after the adjustment of congestive heart failure in our regression model, BUN > 17.7 was still significantly associated with CLI.

So far, there has been an extensive research on the association of uric acid and the development of vascular endpoints in atherosclerosis patients. Uric acid was recently published as a predictor of unhealthy obesity, defined as obesity associated with signs of metabolic syndrome in juveniles and adults.^10^ Liang et al^11^ also showed that serum uric acid was closely associated with elevated aortic sclerosis in adults, independent of conventional cardiovascular risk factors. None of the studies evaluated BUN, an established marker of renal dysfunction, though it seems to reflect hemodynamic changes in atherosclerosis patients. In this context, we investigated BUN and its association with vascular endpoints, especially CLI, in our patients and believe that we are the first to have studied this association in PAOD patients.

BUN has already been studied in CHD patients, whereby an elevated serum BUN level proved to be an independent predictor of long-term mortality in patients after STEMI,^2^ and various studies have questioned the role of BUN in patients with heart failure. In these patients, BUN represents a surrogate marker of the renal response to the systemic hemodynamic changes related to the pathophysiologic mechanisms of heart failure.^12^ Extensive research in this field has established BUN as an independent predictor of mortality in heart failure patients. Elevated BUN is regarded as a predictor for short-term (30 days to 1 year) and long-term (5.3 years) survival in these patients,^13,14^ and was even superior to creatinine and other parameters reflecting renal dysfunction.^13^

Contributing to the research conducted in this field, renal dysfunction was also included in a well-established scoring system for stroke in atrial fibrillation patients.^15^ Recently, the R_2_CHADS_2_ score (impaired renal function, congestive heart failure, hypertension, age ≥75, type 2 diabetes, previous stroke, transient ischemic attack, or thromboembolism [doubled]), was published as a tool to predict stroke and systemic embolism. In this scoring system, impaired renal function defined as creatinine clearance <60 mL/min was included in the CHADS_2_ score (congestive heart failure, hypertension, age ≥75, type 2 diabetes, previous stroke, transient ischemic attack, or thromboembolism [doubled]), and was associated with a high risk for stroke and systemic embolism in atrial fibrillation patients. This score was even further improved by adding BUN, as was published recently.^16^

It is important to note that BUN is influenced by various parameters such as the physiologic decline in renal function with age, which is paralleled by an increase in serum BUN. Furthermore, BUN is also extensively influenced by the coexistence of congestive heart failure, malnutrition, or catabolic state. In our study, however, BUN was still significantly associated with CLI even after the adjustment for the parameters age ≥75 and congestive heart failure in our regression analysis.

CLI is a limb-threatening entity with a prevalence of 20,000 cases/y and an annual incidence of 40/100,000 population.^4^ Its prognosis has improved, thanks to better awareness and treatment options, though the death and amputation rates within 1 year are still high (16.7% and 33.3%, respectively).^17^ In clinical practice, it is often difficult to differentiate PAOD patients according to high and low risk for CLI. Owing to media sclerosis, the ABI might be unreliable, especially in very old patients and those suffering from diabetes.^18^ For these reasons, the serum BUN level might offer an additional marker to discriminate patients at high risk for cardiovascular endpoints including CLI from patients with a lower risk. This does, however, need to be targeted and confirmed in prospective trails.

The major limitation of our study is the retrospective study design and the use of a single blood sample to determine BUN. Furthermore, we did not have any data of echocardiography to further investigate differences in kind or state of congestive heart failure between groups.

We nonetheless were able to show for the first time that BUN > 17.7 can be used to identify atherosclerotic patients at high risk for CLI, especially elderly diabetic patients.

## Acknowledgment

*The authors would like to thank Eugenia Lamont for language editing*.
